# Genetic analyses of the inheritance and expressivity of autonomous endosperm formation in *Hieracium* with different modes of embryo sac and seed formation

**DOI:** 10.1093/aob/mcw262

**Published:** 2017-01-27

**Authors:** Steven T. Henderson, Susan D. Johnson, Joel Eichmann, Anna M. G. Koltunow

**Affiliations:** Commonwealth Scientific and Industrial Research Organization (CSIRO), Agriculture and Food, Waite Campus, Hartley Grove, Urrbrae, South Australia 5064, Australia

**Keywords:** Apomixis, autonomous endosperm, seed, *Hieracium* subgenus *Pilosella*

## Abstract

**Background and Aims** Apomixis, or asexual seed formation, in polyploid *Hieracium* subgenus *Pilosella* species results in clonal progeny with a maternal genotype. An aposporous embryo sac forms mitotically from a somatic cell, without prior meiosis, while embryo and endosperm formation is fertilization independent (autonomous). The latter two developmental components are tightly linked in *Hieracium*. Recently, two plants, AutE196 and AutE24, were identified from two different crosses. Both form embryo sacs via the sexual route by undergoing meiosis, and embryo development requires fertilization; however, 18 % of embryo sacs can undergo autonomous endosperm (AutE) formation. This study investigated the qualitative and quantitative inheritance of the AutE trait and factors influencing phenotype expressivity. An additional focus was to identify the linkage group bearing the *AutE* locus in AutE196.

**Methods** Crosses and cytology were used to examine the inheritance of AutE from AutE24 and AutE196, and to reintroduce apomictic components into AutE plants, thereby changing the ploidy of developing embryo sacs and increasing the dosage of *AutE* loci. Markers from a *Hieracium* apomict linkage map were examined within a backcrossed AutE196 mapping population to identify the linkage group containing the AutE196 locus.

**Key Results** Qualitative autonomous endosperm in the AutE24 line was conferred by a single dominant locus, and the trait was transmitted through male and female gametes in AutE196 and AutE24. Expressivity of the trait did not significantly increase when *AutE* loci from AutE196 and AutE24 were both present in the progeny, within embryo sacs formed via apospory, or sexually derived embryo sacs with increased ploidy. It remains unclear if these are identical loci.

**Conclusions** The qualitative trait of autonomous endosperm formation is conferred by single dominant loci in AutE196 and AutE24. High expressivity of autonomous endosperm formation observed in apomicts requires additional genetic factors. Potential candidates may be signals arising from fertilization-independent embryo formation.

## INTRODUCTION

Seed formation in flowering plants is mediated through sexual and asexual (apomictic) reproduction. Apomixis occurs through a variety of mechanisms and results in progeny that are genetically identical to the maternal parent. Although apomixis is present in > 400 predominantly non-agronomic species, it is absent in cereal crops (Carman, 1997). It has long been recognized that harnessing apomixis for plant breeding has the potential to improve agricultural productivity significantly through rapid preservation of multigenic desirable traits including fixing the yield advantages obtained through hybrid seed (Koltunow *et al.*, 1995; Spillane *et al.*, 2001). However, developing apomixis as a plant breeding technology requires identification of the causal genes and elucidation of the molecular mechanisms which enable female gamete formation without meiosis, fertilization-independent embryo formation and ideally viable endosperm formation (Hand and Koltunow, 2014).

Within the Asteraceae, *Hieracium* subgenus *Pilosella* contains both sexual and apomictic allopolyploid species that are self-incompatible (Fehrer *et al.*, 2007). Developmentally, apomictic *Hieracium* subgenus *Pilosella* species undergo a mitotic form of female gamete formation termed apospory. Specifically, a somatic ovule cell differentiates into an aposporous initial (AI) cell near cells undergoing the meiotic events of female gametogenesis and gives rise to a mitotically derived and therefore chromosomally unreduced embryo sac. Growth of the aposporous embryo sac induces the demise of cells undergoing the sexual pathway. The unreduced egg cell that differentiates in the aposporous embryo sac spontaneously gives rise to an embryo without fertilization. This parthenogenetic or autonomously formed embryo retains the maternal genotype. In *Hieracium*, endosperm formation is also fertilization independent or autonomous, and initiates soon after fusion of two unreduced polar nuclei within the central cell in the embryo sac. Thus, the resultant seed contains endosperm and embryo that lack paternal genomic DNA (Koltunow and Grossniklaus, 2003; Rodrigues *et al.*, 2008). Autonomous endosperm formation is rare in apomicts as fertilization of the central cell is typically required to form viable endosperm in a process termed pseudogamy (Hand and Koltunow, 2014).

Three dominant genetic loci have been identified that control apomixis in *Hieracium*. Two of these are found in apomictic *Hieracium praealtum* (R35), where the *LOSS OF APOMEIOSIS* (*LOA*) locus is activated soon after the sexual events of meiosis are initiated in the ovule, and controls AI cell differentiation, aposporous embryo sac formation and the suppression of the sexual embryo sac formation pathway during aposporous embryo sac expansion (Catanach *et al.*, 2006; Koltunow *et al.*, 2011). The independent *LOSS OF PARTHENOGENESIS* (*LOP*) locus facilitates both autonomous endosperm formation and parthenogenesis in R35 (Catanach *et al.*, 2006). Deletion of either the *LOA* or *LOP* locus in γ-irradiated mutants of R35 results in partial reversion to sexual development. Specifically, deletion of *LOA* prevents AI cell formation and results in the production of meiotically derived embryo sacs, whereas deletion of *LOP* necessitates fertilization for embryo and endosperm development. Deletion of both the *LOA* and *LOP* loci results in complete reversion to sexual reproduction, demonstrating that apomixis superimposes and functionally suppresses the genetically intact sexual pathway in apomictic R35 (Koltunow *et al.*, 2011). The causal apomixis genes at the *LOA* and *LOP* loci have not yet been isolated. *LOA-*linked molecular markers derived from R35 show linkage to the apospory trait in *H. piloselloides* (D36) and *H. caespitosum* (C36) and, in all of these apomictic species, *LOA* is carried on a hemizygous chromosome with a similar structure, suggesting that these three species may share common genic elements at *LOA* (Okada *et al.*, 2011; Kotani *et al.*, 2014).

**Fig. 1.**
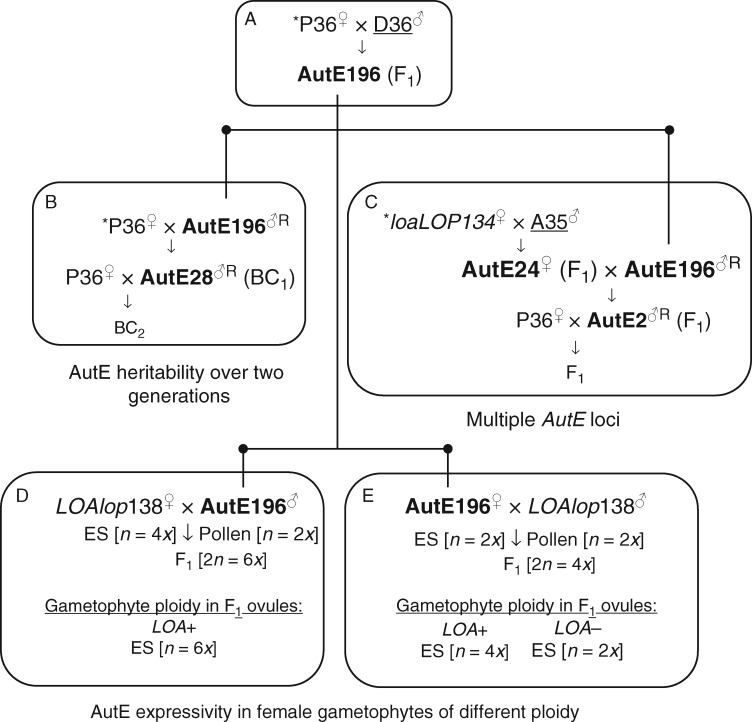
Schema of crosses between tetraploid *Hieracium* subgenus *Pilosella* plants and their applications in this study. (A) Cross between sexual P36 and apomict D36 used to generate the AutE196 autonomous endosperm line. (B) Reciprocal backcrosses of AutE196 to sexual P36 used to test transmission and heritability of the *AutE* locus through male and female gametes over multiple generations. (C) Reciprocal crosses to determine the effects on autonomous endosperm expressivity when the *AutE* loci from the AutE196 and AutE24 lines were combined. (D and E) Reciprocal crosses undertaken to investigate autonomous endosperm expressivity when present in aposporous (*LOA*+; mitotically derived) or non-aposporous (*LOA*–; meiotically derived) embryo sacs of different ploidy. *LOA* segregates within the F_1_ progeny when *LOAlop*138 is the paternal parent. The ploidies of F_1_ plants, embryo sacs (ES) and pollen are indicated in square brackets. *These crosses were reported previously (Ogawa *et al.*, 2013). Apomictic plants are underlined. Plants exhibiting autonomous endosperm only are in bold. ^R^Denotes reciprocal crosses where plants were used as both maternal and paternal parents. Cross orientations used to generate AutE196, AutE24, AutE28 and AutE2 are as depicted. ^♀^Plant used as female parent. ^♂^Plant used as male parent. The *loaLOP*134 and *LOAlop*138 plants are gamma deletion mutants (deleted loci shown in lower case) derived from apomictic R35. The *loaLOP*134 mutant generates reduced embryo sacs via meiosis and undergoes autonomous embryogenesis and endosperm formation. The *LOAlop*138 mutant generates unreduced embryo sacs via mitosis and requires fertilization to generate embryo and endosperm.

Interestingly, segregation of autonomous endosperm formation and parthenogenesis has not yet been observed in crosses between sexual *H. pilosella* (P36) with apomictic R35 or C36 as the apomictic pollen parent (Okada *et al.*, 2011). However, the autonomous endosperm component has been separated from both apospory and parthenogenesis in two hybrid lines, AutE196 and AutE24, derived from other crosses. The AutE196 line was identified among 62 progeny screened from a cross between sexual P36 and apomict D36. AutE24 was isolated amongst 24 progeny screened from a cross between an R35 deletion mutant that had lost *LOA* but retained *LOP* (*loaLOP*134) and apomictic *H. aurantiacum* (A35) (Fig. 1). The autonomous endosperm trait in AutE196 is clearly inherited from the D36 apomict parent in the cross with sexual P36 (Ogawa *et al.*, 2013). However, the parent of origin for the autonomous endosperm trait in AutE24 is unknown as both the *loaLOP*134 and A35 parents have the capacity for autonomous seed development (Koltunow *et al.*, 2011).

Both AutE196 and AutE24 have the same reproductive developmental phenotype, forming chromosomally reduced embryo sacs via the meiotic sexual route, and both require fertilization for embryogenesis. However, in the absence of fertilization in both AutE196 and AutE24, endosperm formation initiates mitotically after fusion of the two polar nuclei within the central cell and subsequently cellularizes, indicating a capacity for functional completion of the endosperm programme. The expressivity of autonomous endosperm formation in AutE196 and AutE24 is approx. 18 % (Ogawa *et al.*, 2013). This is low compared with the high expressivity of autonomous endosperm in apomicts D36 and A35 where autonomous endosperm formation occurs within a chromosomally unreduced aposporous embryo sac in combination with parthenogenesis (Koltunow *et al.*, 2011). The expressivity of autonomous endosperm formation in AutE196 and AutE24 is also lower than in the deletion mutant *loaLOP*134 derived from R35, where *LOP* appears to segregate gametophytically (ie. 1:1 or 50 %) in the meiotically derived embryo sacs, and both embryo and endosperm formation occur concurrently (Koltunow *et al.*, 2011; Ogawa *et al.*, 2013). Viable embryos form following fertilization in AutE196 and AutE24 with frequencies of 77 and 49 %, respectively, indicating that the egg cells are functional and receptive to fertilization (Ogawa *et al.*, 2013).

Analyses of the progeny of a backcross using sexual P36 and AutE196 as female and male parents, respectively, indicated that the autonomous endosperm trait is encoded by a single dominant locus and was transmissible through the male gametophyte (Fig. 1) (Ogawa *et al.*, 2013). The expressivity of the autonomous endosperm phenotype also varied in the backcross population. It is unknown if the autonomous endosperm trait in the AutE196 and AutE24 lines is encoded by the same or different loci (Ogawa *et al.*, 2013). The reasons for the variable and low level of autonomous endosperm formation are also unclear. In an apomict, autonomous endosperm formation typically occurs soon after fusion of the two polar nuclei in the central cell of a chromosomally unreduced embryo sac that has not undergone meiosis. In contrast, both AutE196 and AutE24 form autonomous endosperm in a different developmental context, i.e. within meiotically derived embryo sacs that are chromosomally reduced and do not undergo autonomous embryogenesis. Fusion of the two polar nuclei is also essential before autonomous endosperm formation begins (Ogawa *et al.*, 2013). In other crosses between sexual and apomictic *Hieracium*, apomictic components also exhibit incomplete variable expressivity in the progeny which had been postulated to be due to the influence of unlinked genetic modifiers within the hybrid backgrounds (Bicknell *et al.*, 2000).

This paper describes a series of crosses over multiple generations that examined the transmission of the autonomous endosperm trait through male and female gametes. We also investigated if the presence of multiple *AutE* loci and/or the mode of embryo sac formation, either reduced or unreduced, influenced the expressivity of autonomous endosperm development. Furthermore, we utilized ovule-expressed simple sequence repeat (SSR) markers from a recently developed framework linkage map of the D36 apomict (Shirasawa *et al.*, 2015) to map the *AutE* locus from AutE196.

## MATERIALS AND METHODS

### Plant materials, stock maintenance, glasshouse growth conditions and plant phenotyping

*Hieracium* subgenus *Pilosella* species used in this study are allotetraploid, self-incompatible and are, therefore, obligate outcrossers. The hybrid autonomous endosperm lines AutE196 and AutE24 were generated in a previous study and have lost the capacity for apospory and autonomous embryo formation (Ogawa *et al.*, 2013). Sexual P36 and the R35-derived *LOAlop*138 deletion mutant, which retains capacity for aposporous embryo sac formation but has lost the ability for autonomous seed development, were phenotypically described by Koltunow *et al.* (2011). Stock plants were maintained by vegetative micropropagation in tissue culture to maintain clonal integrity and transferred to soil to permit flowering. Procedures for emasculation, crossing, seed germination and staging of floral capitula, and glasshouse growth conditions were as described previously (Koltunow *et al.*, 1998, 2011). Phenotypic analyses were based on cytological observations of embryo sac, embryo and endosperm development in cleared ovaries at three informative stages of capitula development; stages 6, 8 and 10 (Koltunow *et al.*, 1998, 2011). A minimum of 80 ovules were examined from pollinated and/or unpollinated florets from five different capitula per plant per stage.

### Statistical analyses

The Pearson’s chi-squared (*χ*^2^) test was used for assessing significant differences between categorical variables and the Mann–Whitney U-test (MW) was used for continuous variables where appropriate. For correlation analyses, the Pearson product moment correlation coefficient (*r*) and the Students *t*-test to determine statistical significance were used. The median and interquartile range (IQR) were used to measure the central tendencies and spread, respectively, for the cytologically observed frequencies of autonomous endosperm; immature embryo sacs; eggs and polar nuclei; embryos and endosperm; and aborted embryo sacs, as the prevalence of these features within the ovules of progeny from the various crosses exhibited a skewed distribution.

The R35 deletion mutant *LOAlop*138 exhibits a low-frequency (≤2 %) autonomous endosperm-like trait that is only manifested in secondary chalazal embryo sacs (Koltunow *et al.*, 2011). This phenotype had not been observed in any of the AutE lines or hybrid populations that did not involve crossing with *LOAlop*138. As such, the presence of this specific phenotype in progeny from crosses with *LOAlop*138 was not included in the cytological scoring and statistical analyses of the autonomous endosperm phenotype attributed to AutE.

### Linkage analysis

Linkage analysis used expressed SSR markers designed from ovule-specific transcripts that had been mapped onto a D36 linkage map as reported previously (Shirasawa *et al.*, 2015). SSR markers were resolved on 4 % NuSieve 3:1 TBE agarose (Lonza). Expressed SSR marker oligonucleotides and associated PCR conditions are listed in Supplementary Data Table S1. A P36 × AutE196 BC_1_ backcross population (*n* = 102) with P36 as the maternal parent and AutE196 as the pollen donor was generated previously (Ogawa *et al.*, 2013) and used in a two-way pseudo-testcross mapping strategy. DNA extraction from leaf samples and marker analyses was performed as described previously (Okada *et al.*, 2011).

Simple sequence repeat marker segregation data from the P36 × AutE196 backcross (BC) population were analysed using JoinMap version 4 (Van Ooijen, 2006) using the BC_1_ genotype code. Marker order and genetic distance were determined using a regression mapping algorithm with the following parameters: Haldane’s mapping function, recombination frequency ≤0·3 and LOD (logarithm of the odds) score ≥3·0.

## RESULTS

### Mapping of the autonomous endosperm locus to a linkage group in AutE196

The isolated autonomous endosperm trait in AutE196 was observed in progeny from a cross between sexual P36 and apomictic D36. The framework linkage map for D36 was used as a reference to select a sub-set of SSR markers for delineation of the *AutE* locus location using a P36 × AutE196 BC_1_ mapping population (*n* = 102) (Fig. 1). A preliminary screen was conducted with 20 SSR markers which amplified 21 loci from seven of the 18 D36 linkage groups (data not shown). Initially, the 21 SSR marker loci were evaluated in a sub-set of 43 BC_1_ plants from the P36 × AutE196 cross which consisted of 21 autonomous endosperm plants and 22 non-autonomous plants. Six SSR markers, including the three markers present on the D36 linkage group D08 containing the locus for autonomous seed, exhibited significant segregation distortion from the expected 1:1 segregation ratio (*χ*^2^ ≥12·302, 1 d.f., *P < * 0·001), and linkage to the autonomous endosperm trait could not be reliably assessed. The remaining 15 SSR marker loci from a total of five linkage groups segregated 1:1 (*χ*^2^ ≤2·381, 1 d.f., *P* > 0·1) within the mapping population sub-set. Seven of these 16 marker loci mapped to LGD01, 03, 05, 11 and 15 in the D36 map and showed no significant linkage to the autonomous endosperm phenotype (*χ*^2^ ≤ 3·429, 1 d.f., *P* > 0·06). However, eight of the nine SSR marker loci from LGD07 in the D36 map (HES00092_a, HES04352_b, HES13825_a, HES13730_a, HES08316_a, HES05800_a, HES12196_b and HES05800_c) exhibited linkage to the autonomous endosperm trait and were subsequently analysed in the full mapping population of 102 plants where linkage of these marker loci to the autonomous endosperm trait was confirmed (*χ*^2^ ≥ 4·745, 1 d.f., *P < * 0·03).

**Fig. 2.**
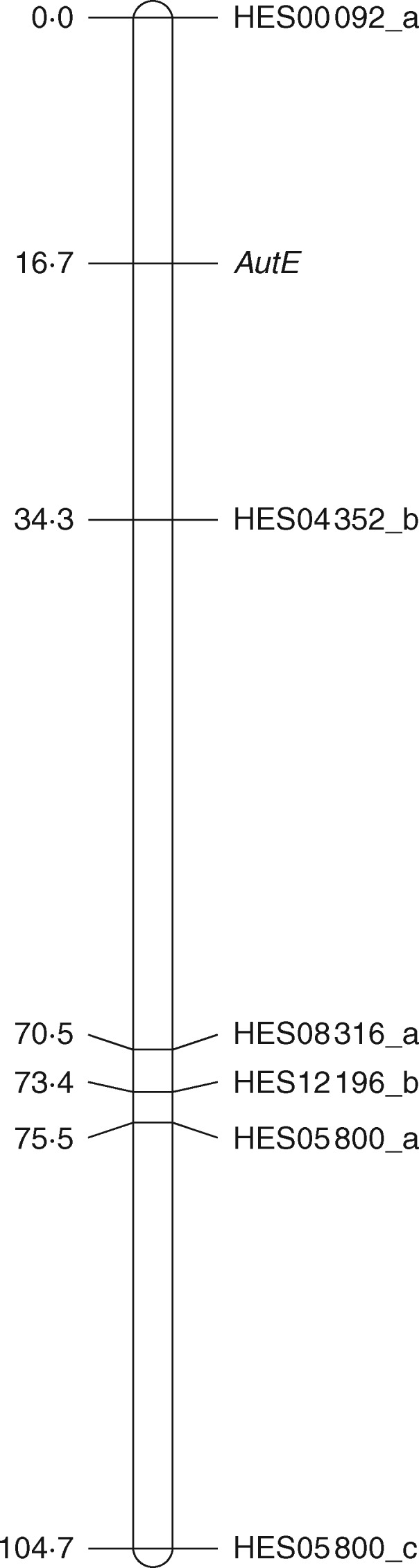
Genetic linkage group in AutE196 encompassing the autonomous endosperm (*AutE*) locus. Six ovule expressed EST-SSR markers grouped together with the autonomous endosperm phenotype. Markers and map distances (cM) are shown to the right and left of the horizontal lines, respectively.

The SSR marker and autonomous endosperm phenotype data for the P36 × AutE196 backcross progeny (*n* = 102) were then analysed using the JoinMap program to generate a linkage map for the *AutE*-bearing linkage group in AutE196. Six of the *AutE*-linked SSR marker loci mapped to a single linkage group (Fig. 2), with the marker order being largely consistent with the D36 LGD07 linkage group map (Shirasawa *et al.*, 2015). The *AutE* locus mapped between the HES04352_b and HES00092_a loci at a distance of 16·7 and 17·6 cM, respectively (Fig. 2). Interestingly, the autonomous endosperm trait did not map to the LGD08 linkage group where the autonomous seed locus and linked markers are located in the D36 apomict (Shirasawa *et al.*, 2015). Reanalysis of the *AutE*-linked markers in the P36 × D36 F_1_ population from which AutE196 was derived had confirmed that none of the *AutE*-linked markers exhibited linkage to the autonomous seed phenotype or associated SCAR (sequence characterized amplified regions) markers (data not shown).

### The autonomous endosperm trait from AutE196 transmits through male and female gametes and is stably inherited over multiple generations

We had previously shown that the autonomous endosperm trait in AutE196 was encoded by a single dominant locus which exhibited 1:1 segregation when transmitted via the reduced male gametophyte (pollen) to the progeny of a P36 × AutE196 backcross (BC_1_) (Ogawa *et al.*, 2013). In a functional tetraploid apomict, the autonomous endosperm and embryogenesis traits initiate from the two fused polar nuclei within a mitotically derived unreduced female gametophyte and an unreduced egg cell capable of parthenogenetic development. As the AutE196 hybrid lacked the capacity for apospory, we were able to examine the autonomous endosperm trait when transmitted through the meiotically reduced female gametophyte. We crossed tetraploids AutE196 and P36 as the female and male parents, respectively, and determined that the autonomous endosperm trait segregated 1:1 (*χ*^2^ >0·348, 1 d.f., *P* = 0·556) within the 46 BC_1_ progeny examined (Supplementary Data Table S2).

We also performed a second series of reciprocal backcrosses (BC_2_) using autonomous endosperm line AutE28, which was a BC_1_ isolate from the P36 × AutE196 cross (Fig. 1). The tetraploid AutE28 line was backcrossed with tetraploid P36 as both the male and female parent, and analyses of these two populations also showed 1:1 segregation of the autonomous endosperm trait (*χ*^2^ <0·474, 1 d.f., *P* > 0·491) (Table S2). Together, the results of the AutE196 and AutE28 reciprocal backcrosses to P36 confirmed that the autonomous endosperm trait derived from D36 can be transmitted without segregation distortion through reduced female and male gametophytes and can be stably inherited in a dominant qualitative manner over at least two backcross generations.

### The AutE28 backcross with sexual P36 exhibits increased embryo sac lethality unrelated to autonomous endosperm

**Fig. 3.**
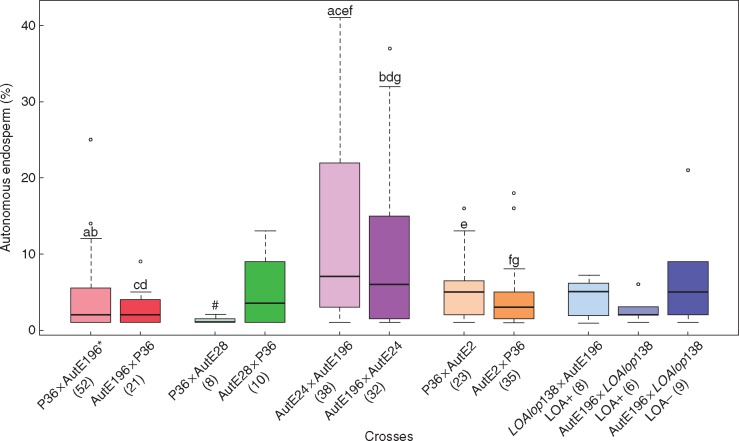
Box-and-whisker diagram for the proportion of ovules with autonomous endosperm in populations from various crosses. Boxes represent first and third quartiles, and the band inside each box indicates the median. Whiskers correspond to 95 % confidence intervals. Outliers are represented by circles. The number of progeny exhibiting the autonomous endosperm trait is shown in parentheses beneath each cross. The statistical significance (*P* < 0·05) of differences in autonomous endosperm expressivities between different crosses was determined by the Mann–Whitney U-test and is shown by paired letters above relevant cross populations. ^#^This cross was not included in quantitative statistical analysis of autonomous endosperm because of a high frequency of embryo sac abortion that was potentially confounding. *This cross was reported previously (Ogawa *et al.*, 2013). LOA+ and LOA– depict progeny that are aposporous and non-aposporous, respectively.

The progeny from the P36 × AutE28 cross (Fig. 1) exhibited a significantly higher rate of embryo sac abortion than observed in the other crosses used in this study (Table 2; median = 56 %; IQR = 34 %; MW < 0·001). The high level of abortion in the P36 × AutE28 cross accounts for the lower frequency of egg cells and polar nuclei observed in this population (Table 2; median = 25 %; IQR = 26 %). Embryo sac abortion was not significantly correlated with the presence or absence of autonomous endosperm formation (*r* = 0·06; *P = *0·800) which segregated within the P36 × AutE28 progeny in the expected 1:1 ratio (*χ*^2^ >0·474, 1 d.f., *P* > 0·491) (Table S2). As such, the P36 × AutE28 population was included in the qualitative segregation analysis of the autonomous endosperm trait. However, the high rate of embryo sac abortion was likely to have adversely affected the expressivity of autonomous endosperm within the P36 × AutE28 population (median = 1 %; IQR = 0·5 %) through reduction in the number of polar nuclei that could have potentially formed endosperm (Table 2; Fig. 3), and was not used for subsequent quantitative comparisons of autonomous endosperm expressivity. The increased number of aborted embryo sacs within the P36 × AutE28 BC_2_ population can most probably be attributed to unknown detrimental factors transmitted and/or expressed gametophytically within this hybrid as the frequency of aborted embryo sacs is substantially lower in the reciprocal AutE28 × P36 BC_2_ cross of the same genetic background.

### Examination of autonomous endosperm formation in plants containing multiple *AutE* loci

The AutE196 and AutE24 lines have different genetic backgrounds (Fig. 1) and it is unknown if the autonomous endosperm loci in AutE24 and AutE196 are alleles of the same gene, or if they are different loci involved in the same, or separate, pathways. We first reciprocally crossed the AutE196 and AutE24 lines which exhibited autonomous endosperm frequencies of 17 and 18 %, respectively (Fig. 1; Table 1). Cytological analyses of the F_1_ progeny of the AutE196 × AutE24 and AutE24 × AutE196 crosses confirmed that the qualitative autonomous endosperm trait segregated in both populations at a ratio of 3:1 (*χ*^2^ ≤3·03, 1 d.f., *P* > 0·08) (Table 2). This segregation ratio is consistent with Mendelian inheritance of two dominant *AutE* loci and suggests that the AutE24 parent also contains a single dominant *AutE* locus that is able to be independently transmitted via male and female gametes.

**Table 1.** Cytological analysis of ovules in stage 10 capitula in non-pollinated parent plant lines

Parent lines	Autonomous endosperm (%)	Immature embryo sacs (%)	Egg cells and polar nuclei (%)	Endosperm and embryos (%)	Aborted embryo sacs (%)	Apospory
P36*	0	0	93	1^†^	6	–
AutE196*	17	1	80	0	2	–
AutE28	25	1	69	0	5	–
AutE24*	18	10	65	0	7	–
AutE2	43	4	46	0	7	–
*LOAlop*138	1^‡^	1	83	0	16	+

* Data published previously (Ogawa *et al*., 2013).

† Embryo and endosperm formation may have been due to incidental cross-pollination as flowers were not isolated.

‡ Endosperm found only in secondary chalazal embryo sacs.

**Table 2.** Cytological analysis of ovules in stage 10 capitula in non-pollinated progeny from various crosses

Cross	Progeny number	Autonomous endosperm^†^ (%)		Immature embryo sacs (%)		Egg cells and polar nuclei (%)		Endosperm and embryos (%)		Aborted embryo sacs (%)	
		Median	IQR	Median	IQR	Median	IQR	Median	IQR	Median	IQR
P36 × AutE196*	102	2 (51)	5	5	7	75	32	0	0	15	23
AutE196 × P36	46	2 (46)	3	6	6	77	26	0	0	17	18
P36 × AutE28	19	1 (42)	1	10	6	25	26	1	1	56	34
AutE28 × P36	21	4 (48)	10	9	4	65	35	0	0	21	24
AutE24 × AutE196	44	7 (86)	20	5	6	70	32	0	0	13	16
AutE196 × AutE24	40	6 (80)	14	5	6	72	19	0	0	10	12
P36 × AutE2	33	5 (70)	5	6	7	73	22	0	0	14	16
AutE2 × P36	45	3 (78)	4	5	7	74	23	0	1	15	12

* Data published previously (Ogawa *et al*., 2013).

† The median of autonomous endosperm is calculated for the percentage of progeny exhibiting the trait (indicated in adjacent parentheses). The frequencies of other reproductive structures were determined for all progeny in each population. These frequencies were similar to the sub-set of plants exhibiting autonomous endosperm within each population.

To determine if the *AutE* loci in AutE196 and AutE24 are homologous alleles of the same gene, we needed to perform a test cross between an isolate containing both *AutE* loci and sexual P36. To this end, we used the AutE2 isolate from the AutE24 × AutE196 cross (Fig. 1; Table 3) as a candidate line postulated to contain both *AutE* loci as it exhibited the highest autonomous endosperm expressivity (43 %) of any isolate in this study (Table 1; Table S2). To confirm whether AutE2 contained two *AutE* loci, we performed reciprocal test crosses with sexual P36 and analysed the segregation of autonomous endosperm within the two populations (Fig. 1). The F_1_ progeny from the reciprocal AutE2 and P36 test crosses exhibited 3:1 segregation (*χ*^2^ ≤ 0·495, 1 d.f., *P* > 0·48) of the autonomous endosperm trait which confirmed that the AutE2 hybrid plant contained two *AutE* loci and also indicated that these *AutE* loci were not homologous alleles of the same gene or closely linked loci on homologous chromosomes (Table 2; Table S2). This result indicates that the *AutE* loci from AutE196 and AutE24 either are different genes or are homeologous alleles, i.e. alleles of the same gene present on independently segregating homeologous chromosomes.

**Table 3.** Cytological analyses of AutE2 ovules at capitula stages 6, 8 and 10, and at stage 10 following emasculation and self-pollination

Capitula stage	Condition	Total ovules	Autonomous endosperm (%)	Immature embryo sacs (%)	Egg cells and polar nuclei (%)	Endosperm and embryos (%)	Aborted embryo sacs (%)
6	Not emasculated	127	15	4	79	0	2
8	Not emasculated	244	20	3	75	0	2
10	Not emasculated	274	43	4	46	0	7
10	Emasculated	122	52	6	41	0	2
10	Self-pollinated	223	47	3	46	0	3

We investigated if the presence of two *AutE* loci from AutE196 and AutE24 had potentially additive effects on expressivity as would be expected if the loci encoded genes involved in separate pathways or were homeologous alleles exhibiting co-dominance. The proportion of progeny from reciprocal crosses between AutE196 and AutE24 which formed autonomous endosperm at higher than parental levels (>18 %), parental levels (between 1 and 18 %) or did not exhibit autonomous endosperm formation (0 %) fit a 1:2:1 ratio (*χ*^2^ ≤ 3·8, 2 d.f., *P* > 0·14) (Table S2). However, the variable expressivity of the autonomous endosperm trait made it difficult to determine if the higher than parental expressivities were due to an additive effect between the two *AutE* loci or if they were a consequence of the complex genetic background inherent in these crosses modulating autonomous endosperm expressivity.

We utilized the reciprocal test crosses between the high expressivity AutE2 line and sexual P36 to investigate the potential modulating effect of genetic background on the expressivity of autonomous endosperm. These crosses generated populations that had genetic backgrounds with greater similarity to the reciprocal backcrosses between P36 and AutE196 or AutE28, which both contained a single *AutE* locus derived from AutE196. The maximum expressivities of autonomous endosperm observed in the P36 × AutE2 (16 %) and AutE2 × P36 (18 %) test crosses were substantially lower than those seen in the AutE24 × AutE196 (43 %) and AutE196 × AutE24 (37 %) crosses (Table S2). The median autonomous endosperm expressivities were significantly lower in the P36 × AutE2 (median = 5 %; IQR = 5 %; MW test: *P* = 0·028) and AutE2 × P36 (median = 3 %; IQR = 4 %; MW test: *P* = 0·001) testcross progeny relative to the AutE24 × AutE196 (median = 7 %; IQR = 20 %) progeny. However, autonomous endosperm expressivity in AutE2 × P36 (MW test: *P* = 0·029) but not P36 × AutE2 (MW test: *P* = 0·183) progeny was significantly lower than in the AutE196 × AutE24 (median = 6 %; IQR = 14 %) progeny (Fig. 3; Table 2). Despite the presence of two segregating *AutE* loci in the AutE2 and P36 reciprocal crosses, the medians of autonomous endosperm showed no significant differences from the P36 × AutE196 (median = 2 %; IQR = 4·5 %; MW test: *P > *0·197), AutE196 × P36 (median = 2 %; IQR = 3 %; MW test: *P > *0·051) and AutE28 × P36 (median = 4 %; IQR = 10 %; MW test: *P > *0·632) crosses which had only one *AutE* allele (Table 2). These results indicated that the presence of two *AutE* loci was not sufficient to increase the frequency of autonomous endosperm formation within meiotically reduced hybrid embryo sacs. Furthermore, it is likely that the different genetic backgrounds had a substantial influence on autonomous endosperm expressivity. As a consequence, we were unable to determine if the *AutE* loci present in AutE24 and AutE196 were different genes involved in the same, or separate, pathways.

### Examination of autonomous endosperm formation in embryo sacs with different modes of formation and ploidy levels

In apomictic *Hieracium* subgenus *Pilosella* species, efficient autonomous embryo and endosperm initiation occurs within an unreduced embryo sac from an egg cell, and from two fused polar nuclei in the central cell, respectively. In contrast, autonomous endosperm formation in unfertilized AutE196 and AutE24 ovules initiates from two fused polar nuclei (*n* = 4*x*) which have arisen within a meiotically reduced embryo sac, and where the meiotically derived egg requires fertilization to initiate embryogenesis. We investigated if the different modes of embryo sac formation and/or ploidy levels affected the expressivity of autonomous endosperm. Crosses were performed between aposporous and autonomous endosperm lines such that autonomous endosperm formation occurred within aposporous embryo sacs in the absence of parthenogenesis. Specifically, reciprocal crosses were performed using the AutE24 or AutE196 autonomous endosperm lines and R35 mutant *LOAlop*138 which had retained *LOA*-mediated apospory but had lost the capacity for autonomous embryogenesis and efficient endosperm formation due to full, or partial, deletion of the *LOP* locus (Koltunow *et al.*, 2011) (Fig. 1D, E).

Reciprocal crossing of the R35 deletion mutant *LOAlop*138 and AutE24 did not produce viable seeds, which was probably due to self-incompatibility mechanisms induced by the large degree of genetic similarity between AutE24 and *LOAlop*138, as the maternal parent of AutE24 was also an R35 deletion mutant (Fig. 1C). Viable seed was generated by reciprocal crosses between the *LOAlop*138 and AutE196 lines which have different genetic lineages (Fig. 1A). Apomictic *Hieracium* subgenus *Pilosella* species produce meiotically reduced (*n* = 2*x*) pollen. Crossing AutE196 and *LOAlop*138 as the pollen recipient and donor, respectively, resulted in the independent segregation of the *LOA* and *AutE* loci in the tetraploid F_1_ progeny (Table 4). Segregation of the autonomous endosperm trait was 1:1 (*χ*^2^ = 1, 1 d.f., *P* = 0·32), whereas the apospory trait and *LOA*-linked markers exhibited segregation distortion with transmission to only 36 % of progeny, which is consistent with previous studies (Okada *et al.*, 2011; Shirasawa *et al.*, 2015). The sub-set of AutE196 × *LOAlop*138 progeny with the *LOA* locus produced predominantly unreduced (2*n* = 4*x*) embryo sacs, whereas the progeny without *LOA*-linked markers did not exhibit clear apospory and produced reduced (*n* = 2*x*) female gametes (Fig. 1E;Table 4).

**Table 4.** Cytological analyses of autonomous endosperm and apospory in ovules in stage 10 capitula from F_1_ progeny derived from reciprocal crosses between AutE196 and the R35-derived *LOAlop*138 mutant

Cross	Autonomous endosperm*^,^^†^ (%)		Progeny number	Apospory	Embryo sac ploidy	Endosperm ploidy
	Median	IQR				
*LOAlop*138 × AutE196	5 (80)	4	10	+	6*x*	12*x*
AutE196 × *LOAlop*138	2 (67)	2	9	+	4*x*	8*x*
AutE196 × *LOAlop*138	5 (56)	7	16	–	2*x*	4*x*

* The median of autonomous endosperm is calculated for the percentage of progeny exhibiting the trait (indicated in adjacent parentheses).

† The low frequency of autonomous endosperm found in secondary chalazal embryo sacs is not included in median calculations as this trait probably originated from the *LOAlop*138 parent (see the Materials and Methods).

The *LOAlop*138 mutant predominantly generates unreduced embryo sacs containing 2*n* = 4*x* egg cells and was used as the maternal parent in a cross with AutE196 as the reduced (*n* = 2*x*) pollen donor to generate hybrid hexaploid (2*n* = 6*x*) F_1_ progeny. All ten progeny examined from this cross were confirmed as aposporous and contained *LOA*-linked markers which indicated that these progeny produced unreduced (2*n* = 6*x*) embryo sacs (Fig. 1D;Table 4). Segregation of the autonomous endosperm trait locus occurred in the F_1_ progeny at an expected 1:1 ratio (*χ*^2^ = 3·6, 1 d.f., *P* = 0·06) (Table 4).

Pairwise comparisons between the *LOAlop*138 × AutE196 (median = 5 %; IQR = 4 %), AutE196 × *LOAlop*138 aposporous (median = 2 %; IQR = 2 %) and the AutE196 × *LOAlop*138 non-aposporous (median = 5 %; IQR = 7 %) F_1_ progeny showed no significant differences (MW test: *P* > 0·30) in the median levels of autonomous endosperm formation (Fig. 3; Table 4). Although autonomous endosperm expressivity was variable, and given the relatively small number of plants examined from these crosses due to technical difficulties, it is likely that a higher ploidy, mitotically derived embryo sac is not sufficient by itself to facilitate high autonomous endosperm expressivity in hybrid populations. Additional factors/signals seem to be required.

## DISCUSSION

### Mapping of the *AutE* locus from *H. piloselloides* (D36)

The autonomous seed phenotype in apomictic *Hieracium* species in subgenus *Pilosella* is comprised of the developmental components of parthenogenesis and autonomous endosperm formation. Inheritance studies of progeny from crosses between sexual P36 as the pollen recipient and apomictic R35, C36 or D36 as pollen donors have shown that the two components comprising autonomous seed development are genetically tightly linked. Parthenogenesis and autonomous endosperm were not separated in the R35- or C36-derived populations, and only one autonomous endosperm isolate was recovered from the D36-derived population (Okada *et al.*, 2011; Shirasawa *et al.*, 2015). *LOP*-linked SCAR markers show tighter linkage to the autonomous seed phenotype in F_1_ hybrid progeny from the P36 × R35 cross relative to the P36 × D36 cross, indicating that there is greater recombination in the vicinity of the autonomous seed locus in D36 relative to R35 (unpubl. data). As AutE196 was derived from D36, we hypothesized that the autonomous endosperm phenotype in the AutE196 line was due to a rare recombination event within the autonomous seed locus that had separated the autonomous endosperm and parthenogenesis loci.

Here we surprisingly found that the *AutE* locus in AutE196 exhibited linkage to seven SSR markers from the D36 LGD07 linkage group rather than to markers from the D36 LGD08 linkage group bearing the autonomous seed locus. It is possible that the autonomous endosperm trait could be due to a spontaneous mutation within AutE196 that is unrelated to the autonomous seed locus in D36. However, the allopolyploid genetic constitution of D36 allows for an alternative hypothesis to explain the uncoupling of autonomous endosperm from parthenogenesis. If LGD08 and LGD07 were homeologous D36 linkage groups then it is possible that a rare homeologous recombination event could have occurred at the autonomous seed locus in D36 which duplicated or translocated the *AutE* locus to a homeologous chromosome that was inherited in the AutE196 BC_1_ offspring. Although meiotic recombination in allopolyploids occurs predominantly between homologous chromosomes, recombination between homeologous chromosomes can also occur and has been shown to facilitate genome rearrangements such as intergenomic duplications and translocations (Gaeta and Pires, 2010). It is currently unknown if LGD07 and LGD08 are actually homeologous linkage groups as there were insufficient SSR markers with multiple polymorphic alleles used to construct the D36 framework linkage map (Shirasawa *et al.*, 2015). Testing this hypothesis requires identification of markers that are closely linked to the *AutE* locus and additional markers to determine if LGD07 and LGD08 are homeologous linkage groups in D36.

### Autonomous endosperm is under single locus control with expressivity modulated by unknown factors in the genetic background

Although the underlying processes that facilitate apomixis are complex, studies in *Hieracium* and other plant species have shown that the qualitative expression of apomixis is often under the control of a small number of Mendelian loci (Ozias-Akins and Van Dijk, 2007). Our genetic analyses of the *AutE* loci from AutE196 and AutE24 in this study have clearly established various qualitative aspects of the autonomous endosperm trait such as non-distorted transmission through male and female gametes; stable inheritance of the autonomous endosperm phenotype from AutE196 over two generations; and that the *AutE* locus from AutE24 appears also to be inherited as a single dominant Mendelian locus. In contrast, the variable expressivity of the autonomous endosperm trait in *Hieracium* makes quantitative genetic analyses challenging.

Reciprocal crossing of the AutE196 and AutE24 lines produced significantly higher median autonomous endosperm expressivities relative to the AutE196 and P36 reciprocal crosses which initially suggested there may have been potential additive effects with two *AutE* loci. However, reciprocal backcrossing of the AutE2 high expressivity line that contained two *AutE* loci with sexual P36 resulted in lower autonomous endosperm expressivities that were not significantly different from the AutE196 and P36 reciprocal crosses which only contained the single *AutE* locus from AutE196. This result indicated that there were modifying factors present in the different genetic backgrounds of the various populations that substantially modulated autonomous endosperm expressivity. As such, it remains unclear if the increased autonomous endosperm expressivities in the AutE196 and AutE24 reciprocal crosses were due to additive effects between the two *AutE* loci, or if the specific genetic background of these crosses was more supportive of autonomous endosperm formation.

Modifying elements present in the genetic background have long been presumed to modulate the expressivity of apomixis components in meiotically derived hybrid populations (Koltunow *et al.*, 1998; Bicknell *et al.*, 2000; Matzk *et al.*, 2005; Ogawa *et al.*, 2013; Hand *et al.*, 2015). The number, identity and function of the putative genetic elements that modulate autonomous endosperm and other apomixis loci are currently unknown. The sexual reproductive pathway in plants is tightly regulated to ensure seed development does not proceed until fertilization. Thus, the variable and typically low autonomous endosperm expressivities in our hybrid *Hieracium* populations may be due to the non-autonomous endosperm parent transmitting specific inhibitory factors to prevent autonomous development from occurring. In addition, or alternatively, the high autonomous endosperm expressivity in the AutE parent lines may be due to a number of supportive, and probably rare, allelic combinations that lose potency when meiotically shuffled. Genetic analyses in intraspecific crosses of *Poa pratensis* has suggested complex, multigenic control of apomixis with expressivity postulated to be regulated by the presence of ‘initiator’ and ‘preventor’ alleles for different apomictic components (Matzk *et al.*, 2005). Quantitative trait analyses in the autonomous endosperm lines would provide insight into the number of genetic modifying factors and the nature of their contribution to the modulation of autonomous endosperm expressivity. In natural populations, the generation of fit plants with specific allelic constitutions that facilitate high expressivity of the various apomixis components would be self-stabilizing through efficient clonal propagation.

### The ploidy state of the female gametophyte and its mode of formation has no apparent effect on autonomous endosperm expressivity in hybrid plants

The processes of autonomous endosperm formation in the AutE196 and AutE24 hybrid lines operate in a different developmental context from that of an apomict due to the absence of apospory and parthenogenesis. Aposporous plants utilize a mode of embryo sac production whereby reprogrammed somatic ovule cells form embryo sacs that have the complete genomic constitution of the maternal plant and have not undergone meiotic recombination. In contrast, the sexual pathway utilizes a different mode of embryo sac formation from functional megaspores that have undergone meiosis-mediated chromosome reduction and recombination. Thus, unfertilized aposporous embryo sacs have twice the ploidy of unfertilized sexual embryo sacs. In addition, although reprogramming of a somatic ovule cell during apospory facilitates the formation of a functional female gametophyte, it is unknown if there are specific adaptions in aposporous and sexual embryo sacs that may be beneficial or detrimental for the expressivity of apomixis, respectively.

The segregation of *LOA* in the AutE196 × *LOAlop*138 cross enabled a direct comparison between the mode of embryo sac formation and ploidy levels in the two population sub-sets. Aposporous progeny generated mitotically derived hybrid embryo sacs (2*n* = 4*x*) with twice the ploidy of meiotically derived hybrid embryo sacs (*n* = 2*x*) generated by non-aposporous progeny. The *LOAlop*138 × AutE196 cross generated hexaploid hybrid progeny (2*n* = 6*x*) through fusion of unreduced clonal egg cells (2*n* = 4*x*) with meiotically derived sperm cells (*n* = 2*x*), and enabled the potential effects of increased gene dosage on autonomous endosperm expressivity to be investigated. No significant differences were observed in the levels of autonomous endosperm between the three progeny classes. This suggests that the mode of embryo sac formation and/or ploidy levels (*n* = 2*x*; 2*n* = 4*x*; 2*n* = 6*x*) are not sufficient by themselves to facilitate increased expressivity of autonomous endosperm in these hybrid populations.

The genetic constitution of each of the F_1_ progeny from the *LOAlop*138 × AutE196 cross included the complete maternal *LOAlop*138 genome. Therefore, the progeny contained all the genetic autonomous endosperm modifiers unlinked to *LOP* that were present in R35, an apomict that exhibited high apomixis expressivity. As there was no significant increase in autonomous endosperm expressivity in the *LOAlop*138 × AutE196 population relative to the AutE196 × *LOAlop*138 population, it appears that the presence of supportive modifiers from R35 were insufficient to facilitate high expressivity of autonomous endosperm in hybrid hyperploidy plants. There are a number of factors potentially associated with the genetic constitution of the hybrid hexaploid progeny that could account for the relatively low expressivity of autonomous endosperm. For example, there may be paternally transmitted inhibitory modifiers that are either dominant to and/or different from the supportive modifiers present in the R35 background. Furthermore, the paternal genetic contribution may have heterochronically perturbed the reproductive pathway and/or resulted in gene dosage effects that might not be conducive to autonomous endosperm formation. An alternative possibility is that high expressivity of autonomous endosperm formation may require the presence of an embryo.

In high expressivity *Hieracium* apomicts, the processes of embryo and endosperm formation occur concurrently without fertilization. Given that the apomicts by-pass fertilization, it may be that dependencies between the processes of embryo and endosperm formation are required to drive efficacy of viable seed formation. None of the progeny from the *LOAlop*138 and AutE196 reciprocal crosses produced embryos due to the absence of parthenogenesis. In sexual plants, embryo and endosperm formation are highly regulated processes (Lafon-Placette and Kohler, 2014). However, the co-ordinating signalling mechanisms between the endosperm and embryo have not been well defined, in particular embryonic signals regulating endosperm development. It has recently been shown that the *CLAVATA3/ESR-RELATED* 19 (*CLE19*) gene has embryo-specific expression and regulates both embryo and endosperm development in *Arabidopsis* (Xu *et al.*, 2015), indicating that embryo-derived factors can influence endosperm development.

### Concluding remarks

The single dominant loci in AutE196 and AutE24 are able to qualitatively confer the autonomous endosperm phenotype. Elucidating this fundamental apomictic process in *Hieracium* requires identification of the gene(s) at the *AutE* loci. To this end, identifying the linkage group bearing the *AutE* locus in AutE196 is an important first step. It is also clear that, like other apomixis components, the expressivity of the autonomous endosperm trait is substantially influenced by unlinked modifying elements present in the genetic background. Identifying these modifiers and their functions presents an additional challenge that will probably need to be addressed in order to realize the ultimate goal of engineering an efficient apomixis platform into crops.

## SUPPLEMENTARY DATA

Supplementary data are available online at www.aob.oxfordjournals.org and consist of the following. Table S1: SSR marker oligonucleotide sequences and PCR conditions. Table S2: cytological analyses of cleared ovules from plants analysed in this study.

## Supplementary Material

Supplementary Data
