# Supplementary material for: Genetic analyses of the inheritance and expressivity of autonomous endosperm formation in Hieracium with different modes of embryo sac and seed formation
Source: Ann Bot. 2017 Jan 27;119(6):1001–10. doi: 10.1093/aob/mcw262 (PMC5604576; doi:10.1093/aob/mcw262)
Supplement: Supplementary Data [file mcw262_Supp.zip › aob-16614-s01.docx]

**Supplementary Table S1** Expressed SSR marker oligonucleotides and PCR conditions

| **Expressed SSR markers** | **D36 Linkage group** | **Forward (5’ – 3’)** | **Reverse (5’ – 3’)** |  |  |
| --- | --- | --- | --- | --- | --- |
| HES02965 | LGD01 | CCACCCATTTGTTTTTGACC | TGCATTTTTAGTAAATTCATGATGCT |  |  |
| HES08977 | LGD01 | TTGACACCAAACCAACGAGA | ACACTCCGCCTTGGACTCTA |  |  |
| HES03616 | LGD01 | TGCTGAAGATGCAACAGGAA | ATTCCCAGGTTATCAAGGGG |  |  |
| HES05636 | LGD01 | TCCGTGAACCAAATCCTATTG | TCGGGAACCTCTTCTACAGC |  |  |
| HES13320 | LGD03 | TCCATCCGTGCATTATCAAA | AGGAGCTAGGGGAGGAGATG |  |  |
| HES00081 | LGD05 | TATTGAGGGCAGACAAACCC | TATCTGTCCCCCAAGCTGTC |  |  |
| HES00092 | LGD07 | AGTGTGTGCGCTTAGTGGTG | TGGTAGATCGCTTTTGCATTT |  |  |
| HES04352 | LGD07 | GATACAACCAGCACCCTCGT | TGTCGCGTTGCTTTAACAAG |  |  |
| HES13825 | LGD07 | TGTTGGGTTTTGTGTGTGCT | CCGTGAATTCATCATCTTTGC |  |  |
| HES13730 | LGD07 | CCCAACGCGTTTTCTAGAGT | GCCGGACTGGTAGGGATTAT |  |  |
| HES08316 | LGD07 | TATACCTGCATCATCGCACC | GCAAAACACACACCAATTTGA |  |  |
| HES05800 | LGD07 | GTCGCTAGGGCTGAAACAAG | GAAAAATCATCCCCAAACGA |  |  |
| HES05867 | LGD07 | TGTGAAATGAATAGACAACCGAA | CGTTTCCTTGAATATATCATCCG |  |  |
| HES12196 | LGD07 | CTCTATGATGACGTGCCGAG | TGGGCTTTGTACCAGATCGT |  |  |
| HES04215 | LGD07 | GCGACCGTGTCATTTTACCT | ACGTCGTTTAACCTATGCCG |  |  |
| HES13395 | LGD08 | GGACCAAACACTCCGACTTT | CGACTGCCGTATTCCTTCTC |  |  |
| HES00444 | LGD08 | AGCAAACCCTCGGTAATTGA | AACTCATGTTGGTTCAGGGG |  |  |
| HES05479 | LGD08 | GTCACAGTCGGAGTCATGGA | AACAGCCGAATCAACATGAA |  |  |
| HES09114 | LGD11 | CTTGGTGGACTTAATGGGGA | GGACTCAATGAAAAGCCTCAA |  |  |
| HES08938 | LGD15 | CTGCAGTCCCATTCCTGATT | GTCTTGGCAGGTAGGCAGAG |  |  |
| PCR conditions: initial denaturation step at 95 ºC for 2 min, followed by 40 cycles of 94 ºC for 30 sec, 55ºC for 30 sec, 72 ºC for 30 sec, and a 2 min final elongation step at 72 ºC. | | | | |  |
